# Basal insulin delivery reduction for exercise in type 1 diabetes: finding the sweet spot

**DOI:** 10.1007/s00125-016-4010-8

**Published:** 2016-06-10

**Authors:** Hood Thabit, Lalantha Leelarathna

**Affiliations:** Wellcome Trust-MRC Institute of Metabolic Science, University of Cambridge, CB2 0QQ Cambridge, UK; Department of Diabetes & Endocrinology, Cambridge University Hospitals NHS Foundation Trust, Cambridge, UK; Endocrinology and Diabetes Research Group, Institute of Human Development, Faculty of Medical & Human Sciences, University of Manchester, Manchester, UK; Manchester Diabetes Centre, Central Manchester University Hospitals NHS Foundation Trust, Manchester Academic Health Science Centre, Manchester, UK

**Keywords:** Basal insulin delivery, Clinical trial, Continuous subcutaneous insulin infusion, Exercise, Hypoglycaemia, Insulin pump, Type 1 diabetes

## Abstract

Exercise poses significant challenges to glucose management in type 1 diabetes. In spite of careful planning and manipulation of subcutaneous insulin administration, increased risk of hypoglycaemia and glycaemic variability during and after exercise may occur as a result of inherent delays in insulin action and impaired counter-regulatory hormone responses. Various strategies to mitigate this issue have been advocated in clinical practice, including ingestion of supplementary carbohydrate prior to exercise, reducing background and pre-meal insulin bolus and performing bouts of resistance/high intensity exercise before aerobic exercise. Insulin pump therapy, considered the most physiological form of insulin replacement for type 1 diabetes allows modulation of basal insulin delivery before, during and after exercise. However uncertainty remains regarding the optimal strategy to reduce basal insulin delivery and its efficacy. In this issue of *Diabetologia*, McAuley and colleagues (DOI: 10.1007/s00125-016-3981-9) report on the impact of a 50% reduction of basal insulin delivery before, during and after moderate-intensity aerobic exercise. Results from this study may contribute to a better understanding of the effects of basal insulin delivery manipulation and may aid in devising therapeutic approaches for glucose management during exercise.

Insulin pump therapy is widely considered to be the most physiological form of insulin replacement for people with type 1 diabetes. It facilitates flexible modulation of basal insulin requirements and allows the user to temporarily change basal insulin delivery before and during periods of enhanced or reduced insulin sensitivity, such as in exercise or intercurrent illness. Despite perceived benefits, the frequency of insulin pump therapy varies widely in different European countries, from below 5% to up to about 25% [1].

Regular physical activity in individuals with type 1 diabetes is associated with several health benefits, including improved fitness and quality of life, improved insulin sensitivity, reduced lipid levels, and lower rates of micro- and macrovascular complications and mortality [2]. Despite the reported benefits and progress in its management, physical exercise remains a challenge for many because of the dysglycaemia that occurs either during or after exercise. Few scientifically rigorous studies have explored how to prevent this from occurring, therefore the evidence-base for patient guidance remains suboptimal. In this issue of *Diabetologia*, McAuley et al [3] report the effects of reducing insulin pump basal rate by 50% 1 h before exercise, on glucose and insulin levels, providing new insights to help inform clinical practice.

In health, exercise causes major changes in glucose production and utilisation, underpinned by rapid changes in circulating insulin, glucagon, catecholamines and other counter-regulatory hormones. Studies in healthy individuals highlight several processes contributing towards glucose turnover during exercise. These include mobilisation of glucose into exercising skeletal muscle via upregulation of insulin-dependent and independent glucose transporters (e.g. GLUT4) [4] and changes in exercise-associated fuel metabolism. The risk of hypoglycaemia associated with the increase in exercise-induced insulin sensitivity is mitigated through reduction of endogenous insulin production, which additionally mediates increased glycogenolysis and fatty acid oxidation.

Exercise poses several challenges to individuals with type 1 diabetes. Manipulating externally administered insulin, with inherent delays in absorption and action can be difficult and requires careful planning. Impaired counter-regulatory responses and inappropriately high insulin levels increase the risk of hypoglycaemia during or after exercise. Fear of hypoglycaemia may cause overcompensation by ingesting excessive amounts of carbohydrate or reducing insulin doses to a greater degree than necessary, leading to high glucose levels during or after exercise. However, a number of alternative strategies have been developed to limit the hypoglycaemia associated with exercise in type 1 diabetes (see text box) [5].

**Table:** 

**Strategies to limit exercise-induced hypoglycaemia in type 1 diabetes**
1. Supplementary carbohydrate intake prior to exercise, without or with reduced dose bolus insulin
2. Reducing pre-exercise basal insulin (this is more difficult for those using insulin injections)
3. Reducing pre-meal insulin bolus for exercise that occurs within 2–3 h of a meal
4. Reducing post exercise basal/bolus insulin to reduce risk of nocturnal or delayed post exercise hypoglycaemia
5. Performing bouts of resistance/high intensity exercise before aerobic exercise
6. Use of novel technology, such as continuous glucose monitoring systems to guide insulin reduction and ingestion of additional carbohydrates

Insulin pump therapy allows the user to temporarily reduce or suspend insulin delivery, providing greater flexibility. This may be particularly useful for individuals undertaking unplanned physical activity and exercise, as well as those who carry out routine physical activity. As a result of delays in the absorption of subcutaneously delivered insulin, however, the effect of insulin delivery rate adjustment is not immediate and, therefore, adjustment prior to exercise related activity is usually recommended. However, clinical advice and guidelines on insulin pump management during exercise varies because of the low level of evidence available.

In spite of current guidelines and diabetes technology such as insulin pump therapy and continuous glucose monitoring, avoiding exercise related dysglycaemia remains a challenge for many individuals with type 1 diabetes. Factors contributing to such complexity include inter- and intra-individual variability in insulin requirements and absorption, timing, exercise type (e.g. aerobic vs anaerobic, start/stop, resistance), intensity and duration of exercise, amount of active insulin on board, antecedent hypoglycaemia, counter-regulatory hormone responses and the changes in insulin sensitivity induced by exercise itself. The effects of varying insulin pump adjustments in type 1 diabetes have been previously reported [6], but uncertainty still exists because of the heterogeneity of experimental conditions and exercise modality used, and reproducibility outside of controlled study environments.

The study by McAuley and colleagues investigated the impact of a 50% reduction of basal insulin delivery 1 h before, during and after moderate-intensity aerobic exercise (30-min of stationary cycling) in the fasting state, in a randomised crossover study design [3]. The results of this study highlighted some interesting findings and relevant key points for healthcare professionals looking after physically active insulin pump users. The first point is what most insulin pump users and diabetes healthcare professionals have experienced in practice: that in conditions where blood glucose levels are low–normal at the start of exercise, reducing basal insulin delivery (even by 50%) does not always mitigate against hypoglycaemia. Further reduction or suspension of insulin delivery coupled with ingestion of carbohydrate beforehand may be necessary, with further carbohydrate supplementation to avoid hypoglycaemia, depending on prevailing blood glucose levels during and after exercise.

The second finding relates to the primary endpoint of the study, namely, circulating free insulin levels. These are increased, albeit transiently, during moderate-intensity exercise, in spite of halving basal insulin delivery 1 h pre-exercise. A plausible explanation is provided by the authors for this finding: increased cutaneous blood flow. Augmented absorption of subcutaneous human and analogue insulin during exercise and at varying ambient temperatures have been reported [7]. Most studies, however, assessed subcutaneous insulin injection rather than continuous insulin pump infusion and so the effects of the latter on insulin pharmacokinetics and pharmacodynamics during exercise have not been well elucidated. The final key finding from McAuley and colleagues is the accelerated reduction in free insulin levels observed immediately post-exercise, postulated to be due to the declining subcutaneous insulin reservoir after exercise and basal insulin delivery reduction. The difference in free insulin levels after exercise was significant in the immediate recovery period (measured by plasma insulin AUC, *p* = 0.028, as shown in the electronic supplementary material [ESM] Table 1 of the article [3]), with subsequent modest increase in plasma glucose level post-exercise compared with rest. Hyperglycaemia during exercise in insulin pump users has been reported previously, although this was following a short period of intense exercise (80% $\overset{\cdot }{V}{\mathrm{O}}_{2 \max }$) and without significant changes to plasma free insulin levels [8].

How does this study influence our understanding of exercise and glucose management in insulin pump users, and what are its implications for clinical practice? The rise in circulating free insulin observed in this study needs further evaluation to increase our understanding of the potential mechanisms associated with this outcome and to determine the clinical implications. It is also important to establish whether the same pharmacokinetic findings occur with different exercise modalities (e.g. in resistance or submaximal exercise) and in various populations (e.g. different age groups and ranging participant characteristics) to determine the generalisability of these study outcomes. The authors hypothesised that, despite increased circulating free insulin levels, the modest rise in glucose with exercise may have been due to the effects of counter-regulatory hormones and that sustainment of glucose levels during recovery may have been due to the decline in insulin levels post-exercise. Combining the glucose level results with data on counter-regulatory hormone and glucose turnover (neither of which were assessed in this study) may help to elucidate the hormonal and metabolic interactions between the three and indicate clinical implications, especially in physically active insulin pump users with impaired counter-regulation. A recent study reported that during intermittent high-intensity exercise in type 1 diabetes without insulin delivery adaption, reduced exogenous glucose requirements may occur secondary to decreased glucose uptake and disposal, rather than increased hepatic glucose output or muscle glycogen utilisation [9]. This potentially novel mechanism warrants further investigation to further our understanding of exercise metabolic adaptation.

Future exercise studies in type 1 diabetes following insulin delivery adjustments should include evaluation of blood glucose levels over longer periods post-exercise since delayed hypoglycaemia, particularly overnight, remains a clinical concern. Such studies should recruit participants with impaired hypoglycaemia awareness and antecedent hypoglycaemia events, who have an increased risk of hypoglycaemia. In addition to pre-exercise insulin delivery interventions, studies comparing or combining this strategy with novel hypoglycaemia avoidance strategies, such as threshold/predictive suspended insulin pump management [10], intermittent high intensity exercise or acute caffeine ingestion [11], may help uncover management approaches best suited for individuals with a variety of characteristics and requirements. A novel approach, using the artificial pancreas, has reported improvements in glycaemic control during free daily living home studies [12], but further technological development is needed to optimise its efficacy during exercise. Gathering evidence from well-designed studies reflecting ‘real world’ conditions may ultimately help healthcare professionals to better understand and safely optimise therapeutic management during exercise.
